# Isolation and Characterization of a Novel Dicistrovirus Associated with Moralities of the Great Freshwater Prawn, *Macrobrachium rosenbergii*

**DOI:** 10.3390/ijms17020204

**Published:** 2016-02-02

**Authors:** Xiaoyi Pan, Zheng Cao, Junfa Yuan, Zhengli Shi, Xuemei Yuan, Lingyun Lin, Yang Xu, Jiayun Yao, Guijie Hao, Jinyu Shen

**Affiliations:** 1 Key Laboratory of Healthy Freshwater Aquaculture, Ministry of Agriculture, Key Laboratory of Fish Health and Nutrition of Zhejiang Province, Zhejiang Institute of Freshwater Fisheries, Huzhou 313001, China; cz0305@163.com (Z.C.); yuanxuemei_2008@163.com (X.Y.); linly0531@163.com (L.L.); fionxu126@163.com (Y.X.); yjy0913lxl@126.com (J.Y.); 13867253205@163.com (G.H.); 2 Department of Aquatic Animal Medicine, College of Fisheries, Huazhong Agricultural University, Wuhan 430070, China; jfyuan@mail.hzau.edu.cn; 3 State key Laboratory of Virology, Wuhan Institute of Virology, Chinese Academy of Sciences (CAS), Wuhan 430071, China; zlshi@wh.iov.cn

**Keywords:** *Macrobrachium rosenbergii*, dicistrovirus, *Aparavirus*, taihu virus, larval mortality syndrome

## Abstract

The giant freshwater prawn, *Macrobrachium rosenbergii*, is an economically important crustacean and is farmed in many countries. Since 2009, a larval mortality syndrome of *M. rosenbergii* has broken out and spread widely in the main breeding area, including Zhejiang, Jiangsu, Guangxi, and Guangdong Provinces in mainland China. A novel virus, named *Macrobrachium rosenbergii* Taihu virus (MrTV), was isolated from the moribund larvae and was determined to be the causative agent of the *M. rosenbergii* larval mortality syndrome by experimental infection. Further genomic sequencing suggested that the MrTV genome is monopartite, 10,303 nt in length, and dicistronic with two non-overlapping open reading frames (ORFs) separated by an intergenic region (IGR) and flanked by untranslated regions (UTRs). Phylogenetic analysis using the full-length genomic sequence and the putative amino acid sequences of the capsid protein revealed that MrTV was more closely related to the taura syndrome virus (TSV) than to any other viruses. According to these molecular features, we proposed that MrTV is a new species in the genus *Aparavirus*, family Dicistroviridae. These results may shed light on controlling larval mortality syndrome in *M. rosenbergii*.

## 1. Introduction

The giant freshwater prawn, *Macrobrachium rosenbergii*, is an economically important crustacean and is farmed in many countries. According to the United Nations Food and Agriculture Organization (FAO) (2009), the total production of cultured *M. rosenbergii* reached 207,749 tons in 2008, of which Asia produced 127,627 tons. China is the largest farming country of *M. rosenbergii*. To date, several viral diseases have been documented in *M. rosenbergii*. One is due to a parvo-like virus affecting the digestive tract [1]. The second viral disease is white tail disease (WTD) caused by *M. rosenbergii* nodavirus and is associated with postlarvae mortality in *M. rosenbergii* [2,3]. In addition, infectious hypodermal and hematopoietic necrosis virus (IHHNV), macrobrachium muscle virus (MMV), and hepatopancreatic parvovirus (HPV) have also been described in cultures of *M. rosenbergii* [4,5,6]. Covert mortality nodavirus (CMNV), associated with the covert mortality disease of shrimp were also detected in the cultured *M. rosenbergii* ([7] and personal communication).

In 2009, a larval mortality syndrome of *M. rosenbergii* broke out in a *M. rosenbergii* hatchery located in Huzhou, Zhejiang Province, China. Afterwards, similar diseases were found in other main breeding areas of *M. rosenbergii*, including Zhejiang, Jiangsu, Guangxi, and Guangdong Provinces in China. This disease mainly threatened *M. rosenbergii* larvae, especially zoeal stage V. The clinical signs of the diseased larvae include moulting obstacles, red shed shell, decreased response to stimuli, sinking to the bottom, and eating difficulties. In general, the mortality rate of this disease ranges from 80% to 90%, and the peak mortality rate occurs in the seven-day-old larvae.

To investigate the causative agent of the larval mortality syndrome of *M. rosenbergii*, efforts were made to isolate the potential pathogenic bacteria from moribund larvae initially. Different bacteria were isolated from different sources of larvae, but similar clinical signs could not be replicated by experimental infection, which suggested that bacteria were not the main pathogen. Therefore, we analyzed viral pathogens using classical methods of virology. Hence, we reported a novel virus, which was isolated from the diseased larvae on the south bank of Taihu Lake and provided the molecular characterization of this novel virus, named *Macrobrachium rosenbergii* Taihu virus (MrTV).

## 2. Results

### 2.1. Isolation of an Unknown Virus

**Figure 1:**
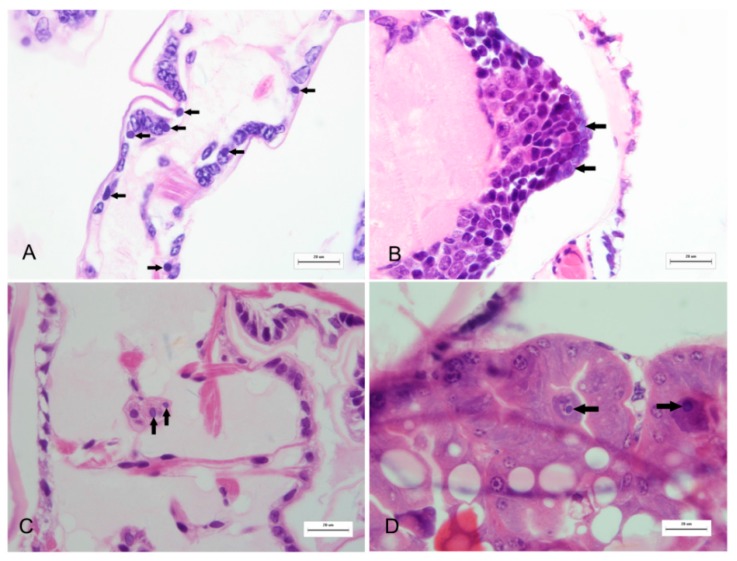
Illustration of viral inclusion bodies in the histological sections of diseased *M. rosenbergii* larvae. Pale to dark basophilic, intracytoplasmic inclusion bodies with 2.8 to 4.0 μm in diameter are observed in a number of cells (indicated by arrows) in appendage epithelial tissues (**A**); ganglion (**B**); connective tissue (**C**); and hepatopancreas (**D**). All H and E. Bars = 20 μm.

To explore the potential viral pathogen of *M. rosenbergii* larval mortality syndrome, moribund larvae were collected for viral examination according to standard techniques. First, the collected larvae were processed for histopathological examination after fixed in 10% Bouin’s fixative using standard procedure, consisting of paraffin embedding, sectioning, and hematoxylin and eosin (H and E) staining. Histopathological results showed that cytoplasmic viral inclusions were observed in the cuticle epithelium, collective tissue, ganglion from sick larvae (Figure 1), but not observed in the tissue of healthy larvae. These viral inclusions were generally discrete, pale to darkly basophilic (with H and E staining) and from 2.8 to 4.0 μm in diameter.

**Figure 2:**
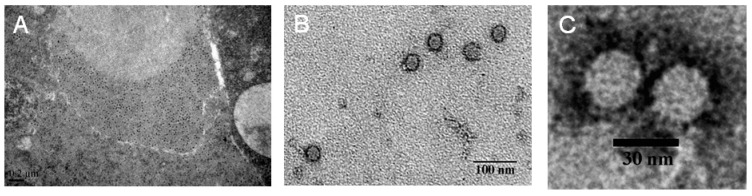
Examination of virus particles by transmission electron microscope (TEM). (**A**) Numerous hexagonal, non-enveloped virus particles observed in the cytoplasm. Bar = 0.2 μm; (**B**) purified virus particles observed by electron microscopy. Bar = 100 nm; and (**C**) higher magnification of purified virus particle indicated in (**B**). Bar = 30 nm.

Then, the diseased larvae were used for ultrathin sections and examined under a Hitachi H-7000FA transmission electron microscope (TEM) at 75 kV after double-staining with uranyl acetate and lead citrate. Mass virus-like particles approximately 25–29 nm in diameter were observed to be interspersed within the cytoplasm of connective cells (Figure 2A).

To isolate the observed unknown virus, the collected moribund larvae were ground and used for virus isolation by sucrose density gradient centrifugation. Transmission electron microscopy examination suggested that numerous non-enveloped viral particles were located in the 40% sucrose solution density gradient. The mean size of the viral particles was 25–29 nm in diameter (Figure 2B,C).

### 2.2. The Purified Virus Is an Unknown RNA Virus

Random-PCR and sequencing were used to identify the unknown virus. Several fragments of 500 base pair (bp) in length were obtained using the Random-PCR method. Sequencing and blasting results showed that the obtained fragments of the unknown virus shared similarities with TSV, which was identified as a member of Dicistroviridae. From these results, we implied that the unknown virus was related to dicistroviruses.

**Figure 3:**
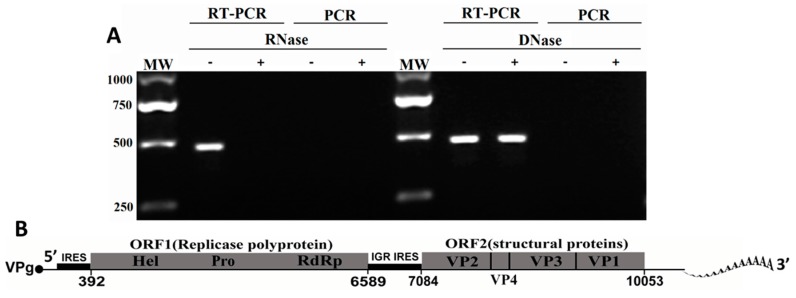
Characterization of the MrTV. (**A**) Sensitivity of MrTV RNA to single strand-specific RNase A or DNase. RNase A-digested (+), DNase-digested (+), or Control (−) nucleic acids of the viral particles prepared. RT–PCR and PCR were conducted with specific primer pairs; *M*w, molecular weight; and (**B**) schematic diagram of the genome organization of MrTV indicated in the orientation of 5′ to 3′. The genome has a small peptide covalently linked to the 5′ end (genome-linked virus protein, VPg) and a 3′ polyadenylated terminus. Two open reading frames with conserved protein domains (RNA helicase, Hel; cysteine protease, Pro; RNA-dependent RNA polymerase, RdRp) are indicated. Two conserved internal ribosome entry site (IRES) located in the non-translated region are shown.

To confirm the genomic type of this virus, the total nuclear acid was extracted from the purified viral particles and pre-treated with RNase or DNase. Subsequently, the treated nucleic acid was used for RT-PCR or PCR with a special primer pair. As shown in Figure 3A, an expected band approximately 472 bp in length was observed through RT-PCR methods both with and without pre-treatment with DNase, while no expected band was obtained when the nucleic acid of the unknown virus was pre-treated with RNase. The expected band was not observed through direct PCR methods for viral nuclear acids that were pre-treated or not. From the results, we suggest that the isolated virus from *M. rosenbergii* larvae was a RNA virus, and it was designated *M. rosenbergii* Taihu Virus (MrTV) according to the sampling location.

### 2.3. Genome Characteristics of MrTV

Based on the partial genomic sequences obtained by random PCR, special primers were designed and used to amplify the unknown genomic region of MrTV. Except for the termini, the full-length genome of MrTV was assembled by seven overlapping cDNA clones. The terminal sequences of the Taihu virus was obtained by two successive 5′ RACE reactions and one 3′ RACE reaction. The full-length genome of the MrTV comprises 10,303 nt (GenBank accession number: HQ113110 or NC 018570). The overall nucleotide composition of MrTV is 40.5% in G+C.

Figure 3B shows the genome structure of MrTV. The RNA genome of MrTV is dicistronic with two non-overlapping ORFs separated by an intergenic region (IGR) and flanked by UTRs. The 5′-proximal ORF (ORF1, nt 392–6589) encodes a polyprotein precursor of 2066 amino acids (aa), flanked by a 5′ UTR of 391 nt and a 3′ UTR of 239 nt and poly (A) tail. The components of ORF 1 contain a RNA helicase (Hel), a RNA-dependent RNA polymerase (RdRp), and a cysteine protease. The 3′-proximal ORF (ORF2, nt 7084–10,053) encodes the capsid protein of 990 amino acids in length. These two ORFs account for 89.1% of the genome. The remaining region contains a 5′ terminus (391 nt), IGR (494 nt), and 3′ terminus (239 nt). In summary, the genomic structure of MrTV was highly similar to that of Dicistroviridae.

### 2.4. MrTV in Relation to Other Dicistroviruses

To identify the classification of MrTV, the genomic sequence was aligned with those of dicistroviruses that were available in the GenBank database. Sequence analysis results suggested that the genome of MrTV shared 32.7% and 34.5% identity with species from two genera in the family Dicistroviridae, namely, cricket paralysis virus (CrPV,) and acute bee paralysis virus (ABPV), respectively (Table 1). The MrTV shared 58.7% and 55.1% identity with the two dicistroviruses isolated from crustaceans, namely, taura syndrome virus (TSV) and mud crab dicistrovirus (MCDV), respectively.

The deduced amino acid sequence of RdRp encoded by MrTV was aligned with other members of the dicistroviruses that were available in the GenBank database. As shown in Table 1, the Taihu virus shared 86.7%, 79.6%, 45.2%, and 44.6% identity with TSV, MCDV, CrPV, and ABPV, respectively. The deduced amino acid sequence of the capsid protein (ORF2) encoded by MrTV was aligned with other members of the dicistroviruses that were available in the GenBank database. The results indicated that the MrTV shared 71.7%, 60.4%, 18.3%, and 18.9% identity with TSV, MCDV, CrPV, and ABPV, respectively (Table 1).

**Figure 4:**
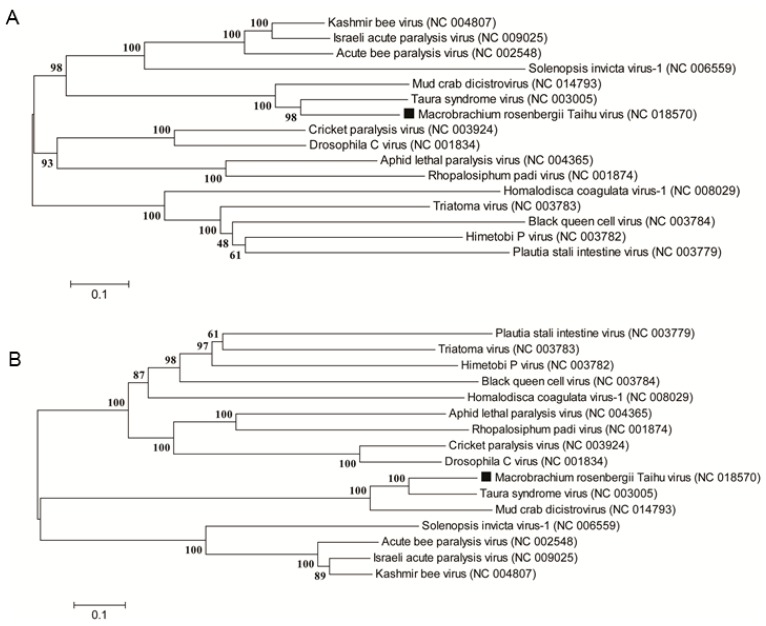
Phylogeny of the MrTV and other dicistroviruses. Phylogenetic trees based on the full-length genomic sequences (**A**) and the deduced amino acid sequences of the capsid protein (**B**) were constructed using the neighbor-joining method with 1000 bootstrap replicates under the parameter of complete deletion and poisson model using MEGA 6.0. The numbers at the branch nodes represent the bootstrap confidence levels of the 1000 bootstrap replications obtained. Bar, 0.1 amino acid substitutions per site. The black aquares indicate MrTV.

To identify the relationship of the MrTV in dicistroviruses, phylogenies were constructed based on the full-length genome of the MrTV and all available dicistroviruses. Phylogenies suggest that MrTV shows close phylogenetic relationship with TSV and clusters other Aparaviruses (Figure 4A). When the deduced amino acid sequences of capsid proteins were used to construct the phylogenetic tree, similar patterns of phylogenetic positions were observed (Figure 4B). These results suggested that the MrTV formed a cluster with Aparavirus and was distantly related to Cripavirus.

**Table 1:** Sequence similarity of the full-length genome, deduced RdRp, or structural protein of the MrTV to other dicistrovirues. The reference sequences of dicistrovirues were indicated in Figure 4.

Virus	Acronym	Genome (%)	RdRp (%)	ORF2 (%)
**Genus: *Cripavirus***				
**Cricket paralysis virus ^1^**	CrPV	32.7	45.2	18.3
Aphid lethal paralysis virus	ALPV	30.6	29.4	17.7
Black queen cell virus	BQCV	35.9	32.0	18.0
Drosophila C virus	DCV	34.0	41.0	17.3
Plautia stali intestine virus	PSIV	34.0	35.3	16.2
Rhopalosiphum padi virus	RhPV	30.3	32.4	19.1
Triatoma virus	TrV	35.1	34.3	19.4
Homalodisca coagulata virus-1	HoCV-1	34.6	37.0	17.7
Himetobi P virus	HPV	31.8	37.2	16.8
**Genus: *Aparavirus***				
**Acute bee paralysis virus ^1^**	ABPV	34.5	44.6	18.9
Taura syndrome virus	TSV	58.7	86.7	71.7
Kashmir bee virus	KBV	34.2	44.2	18.5
Solenopsis invicta virus-1	SINV-1	29.1	43.8	17.5
Israeli acute paralysis virus	IAPV	34.5	45.7	19.1
**Unclassed genus**				
Mud crab dicistroviruses	MCDV	55.1	79.6	60.4

^1^ represents the type species.

**Figure 5:**
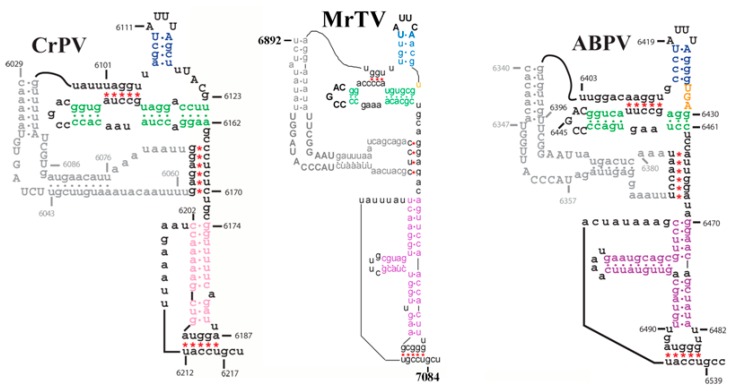
Structure of the intergenic region (IGR) internal ribosome entry site (IRES). The structure of the IGR-IRES in MrTV genome was predicted using the Mfold web server (http://mfold.rna.albany.edu//?q=mfold/RNA-Folding-Form). MrTV had a bulge sequence (UGGAUACCCAU and UAAGGCUU) in the IGR IRES. Additional stem loop structures and nucleotide sequences in the bulge regions were present in MrTV (indicated in bold). The red stars and dots indicate nucleotide interactions in pseudoknots and stems. The different color styles correspond to the different stems. CrPV (cricket paralysis virus; type species of *Cripavirus*), ABPV (Acute bee paralysis virus; type species of *Aparavirus*).

Sequence analysis indicated that the MrTV included a bulge sequence (UGGAUACCCAU and UAAGGCUU) in the IGR-IRES region (Figure 5), which was conserved in aparaviruses. The former was different from ABPV with a nucleotide. Additionally, there was an additional stem loop present in the 3′ region of IGR-IRES, which was seen in aparaviruses, but not in cripaviruses.

In summary, MrTV is related to the family Dicistroviridae and can be classified in the genus Aparavirus according to the current classification criterion.

### 2.5. MrTV Is the Causative Agent of the Disease

**Figure 6:**
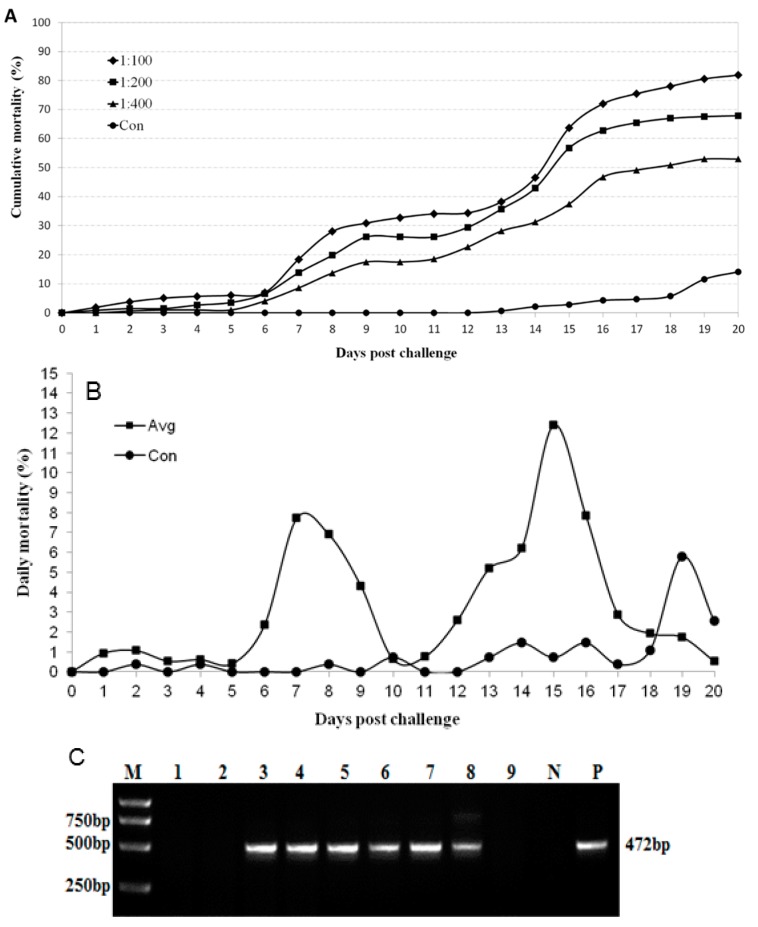
Experimental infection of *M. rosenbergii* with MrTV. Curves of cumulative mortalities in each group (**A**) or daily mortalities in the three infected groups on average (**B**) were counted at various times during the post-immersion challenge. Three infected groups with different concentration filters (1:100, 1:200, and 1:400) of tissue homogenates were indicated, respectively. The control group was immersed in phosphate-buffered saline (*n* = 200). Virus infections from dead larvae in the tested groups (at days 3, 7, and 15) or the control group (at days 0 and 19) were confirmed by RT-PCR (**C**): M, DNA ladder; 1, *Chirocephalus*; 2, zero-day in control group; 3–5, seven-day for three infected groups; 6–8, fifteen-day for three infected groups; 9, control group at day 19; N, negative control; P, positive control.

To confirm the pathogenicity of MrTV for *M. rosenbergii* larvae, healthy larvae were infected with viral suspensions in a series dilution. As shown in Figure 6A, *M. rosenbergii* larvae infected with the filtrate from diseased larvae began to die at one day post-infection, and the cumulative mortalities reached 53%–82% within 20 days. We also observed that the test group infected with higher concentrations of the viral suspension showed higher cumulative mortalities than other test groups. The control group began to die at 13 days, and the cumulative mortality was 14% within 20 days due to larval metamorphosis. It was interesting to find that there were two peak times of death in the infected groups, days 6–9 and 12–17 post infection, when the mean daily mortality in all infected groups was considered (Figure 6B). The clinical signs and histopathological changes of the experimentally-infected larvae were similar to those in the naturally-infected larvae described above. No apparent pathological signs were observed in the control group, although a lower peak mortality appeared at Day 19.

The dead larvae and chirocephalus were collected and analysed using a pair of primers targeting the MrTV through RT-PCR methods. As shown in Figure 6C, MrTV was detected in the dead larvae sampled from the three infected groups at days 7 and 15 post-infection. No band was amplified from the larvae before infection, the chirocephalus, or the dead larvae in the control group at day 19. In summary, MrTV was the causative agent of the *M. rosenbergii* larval mortality syndrome.

## 3. Discussion

Dicistroviruses are members of a rapidly growing family of picoranvirus-like RNA viruses, which are named as the *Dicistroviridae* [8,9]. Dicistroviruses are pathogenic to beneficial arthropods, such as honey bees, shrimp, and insect pests of medical and agricultural importance [10,11,12,13]. Currently, only two species of dicistroviruses have been reported in crustaceans. One was the TSV in *Penaeus vannamei* shrimp, which was first reported in Ecuador in 1991 [14]. The other was the mud crab dicistrovirus-1 (MCDV-1), which caused 100% mortality in crabs [15]. This study reported a third member of dicistroviruses, MrTV, in crustaceans that was confirmed to be the causative agent of the larval mortality syndrome in *M. rosenbergii*.

In dicistrovirus, the RNA genome is monopartite and dicistronic with two non-overlapping ORFs separated and flanked by UTRs. Genomic structural analysis revealed that the MrTV genome was arranged in this typical organization. In MrTV, two separated ORFs, flanked by UTRs, were identified. The 5’-proximal ORF encode a replicase protein and the 3’-proximal ORF encode a capsid polyprotein. Phylogenetic analysis using the full-length genome sequence, the putative amino acid sequences of the capsid protein and the RdRp (or polyprotein of ORF1) revealed that MrTV was more closely related to TSV than to any other viruses (Figure 4 and Figure S1). According to these two features, MrTV could be a novel member of *Dicistroviridae*.

Dicistroviridae contains two genera, *Cripavirus* and *Aparavirus*, which can be distinguished by the conserved IGR-IRES sequence. The *Cripaviruses* have a conserved bulge sequence (UGAUCU and UGC) in the IGR IRES, while the *Aparavirus*es have a different bulge sequence (UGGUUACCCAU and UAAGGCUU) [16,17]. Additional stem loop structures and nucleotide sequences in the bulge regions are present in *Aparavirus*es. Upon comparing the IGR IRES sequences of the MrTV with other dicistrovirues, the results indicated that MrTV contains the following bulge sequence: UGGAUACCCAU and UAAGGCUU. Considering the identity of the deduced structural protein less than 72% (Table 1), we proposed that MrTV is a new species in the genus *Aparavirus*.

Viral diseases are a threat to the aquaculture industry of *M. rosenbergii*. An emerging viral disease caused by MrTV was identified, which caused huge losses to the cultivation of *M. rosenbergi*. In accordance with clinic observations, experimental infections suggested that MrTV was lethal to larvae of *M. rosenbergii* and that its mortality ranged from 53% to 82% within 20 days. However, no mortality was observed when adult *M. rosenbergii* were injected with MrTV, although the viral RNA was detected in gill tissue at 10 days post-infection (data not shown). These results indicated that adult shrimp can carry MrTV and not be affected. Although the transmission of MrTV has not been confirmed, we suspected that vertical transmission of MrTV was conceivable. Additionally, a preliminary survey suggested that the wild *M. rosenbergii* collected in natural water bodies was negative for MrTV. These data suggest that breeding of specific pathogen-free *M. rosenbergii* stock is a feasible measure to prevent MrTV.

Interestingly, two mortality peaks of larvae (7 and 15 days) were observed after immersion with MrTV (Figure 6B), which may be associated with the special developmental stages of larvae. According to larval development, of *M. rosenbergii* endure exposure to eclosin 11 times from larvae to juvenile shrimp (ZI–ZXI). During each time, they are fragile and sensitive to changes in the water environment or other stresses, especially in the larvae of seven days and 15 days. The former is during zoeal stage V and begins by changing the diet from *chirocephalus* to egg custard, while the latter is the key time point for *M. rosenbergii* to develop into the post-larval stage from zoeal stage XI and is accompanied with changes in swimming behavior and diet. The MrTV infection made the situation worse in these two stages, which was in accordance with the clinical observations. In fact, the larval mortality syndrome disease was called the “disease of seven days” in the breeding process of *M. rosenbergii*.

In conclusion, MrTV, a lethal virus of *M. rosenbergii*, was isolated and characterized as a novel member of the Dicistroviridae according to its genomic features. This expands the family of Dicistroviridae and may shed light on controlling the larval mortality syndrome of *M. rosenbergii*.

## 4. Materials and Methods

### 4.1. Larvae of M. rosenbergii

The diseased larvae were collected from hatcheries located in the Huzhou district, Zhejiang Province, China. The larvae were washed in sterile saline, transported to the laboratory on dry ice, and stored at −80 °C for further study.

One-day-old healthy larvae of *M. rosenbergii* were purchased from a hatchery in Huzhou, Zhejiang Province, China. The larvae were maintained in 50 × 38 × 23 cm^3^ disinfected tanks and fed *chirocephalus* three times per day.

### 4.2. Virus Isolation, Purification, and Examination by Electron Microscopy

Approximately 20 g of moribund larvae samples were collected for virus isolation. After thawing, the larvae were weighed and ground by mortar and pestle with beads of alundum in TN buffer (50 mM Tris–HCl, 100 mM NaCl, pH 7.4) in a 1:10 proportion. The tissue homogenates were centrifuged at 10,000× *g* for 15 min. The supernatant was collected and filtered through a nylon net (400 mesh) for virus purification. Viral particles in the supernatant were concentrated by centrifugation through a 30% sucrose cushion at 184,000× *g* for 3 h at 4 °C using a Ty70 rotor (Beckman, Brea, CA, USA). Next, the pelleted viruses were dissolved in TN buffer and further purified by discontinuous sucrose gradient centrifugation (30%–60%) at 114,000× *g* for 3 h at 4 °C using a Ty90 rotor (Beckman). The band at approximately 40% sucrose was collected and centrifuged at 114,000× *g* for 3 h after being washed with TN buffer. The pelleted viruses were dissolved with TN buffer, and aliquots were stored at −70 °C.

Purified viruses were checked by electron microscopy using Formvar- and carbon-coated copper grids (200 mesh) (Zhongjingkeyi Inc., Beijing, China), negatively stained with 2% phosphotungstic acid (pH 7.0), and examined at 75 kV with a Hitachi H-7000FA transmission electron microscope (Hitachi, Tokyo, Japan).

### 4.3. Extraction of Viral Genome or Total RNA

The viral genome was extracted for characterization. Briefly, viral suspensions were digested with 200 μg/mL proteinase K in TE buffer (10 mM Tris-HCl, 10 mM EDTA, pH 8.0) containing 0.5% sodium dodecyl sulphate (SDS) at 37 °C for 1 h. The viral genome was extracted with phenol/chloroform/isoamyl alcohol (25:24:1, *v*/*v*/*v*) and chloroform/isoamyl alcohol (24:1, *v*/*v*) and then precipitated with 2.5 volume of absolute ethanol after addition of 0.3 M sodium acetate (final concentration) at −20 °C for 2 h, followed by washing with 75% ethanol and dissolving as above. Then, the viral genome was divided into three parts, and two parts were digested with RNase A (10 ng/μL) or DNase I (0.2–0.3 U/μL) at 37 °C for 30 min. The remaining part was used as a control. The treated viral genome was stored at −70 °C.

Total RNA was extracted from whole larvae with TRIzol reagent (Invitrogen, Carlsbad, CA, USA) according to the manufacturer’s protocol. The final RNA was resuspended in 40 to 50 μL of diethy pyrocarbonate (DEPC) water and stored at −70 °C.

### 4.4. Random PCR Procedure

A random PCR procedure covering both RNA and DNA was used to identify the unknown viral genome [18,19]. Briefly, purified virus particles were treated with 10 ng/μL RNase A and 100 U DNase I (Promega, Madison, WI, USA) in a total volume of 140 μL at 37 °C for 30 min. A QIAamp Viral RNA Mini Kit (Qiagen, Hilden, Germany) was used to extract the viral nucleic acid (either DNA or RNA) according to the manufacturer’s protocol. cDNA synthesis was performed by incubating the extracted viral nucleic acid at 90 °C for 5 min, followed by quenching on ice for 2 min. A 20-μL reaction mixture containing the following was prepared: 10 pmol of universal primer-dN6 (5’-GCCGGAGCTCTGCAGAATTCNNNNNN-3’), 10 μL of denatured nucleic acid, 0.6 mM aliquots of each deoxynucleoside triphosphate (dNTP), 20 U RNase inhibitor, and 200 U M-MLV reverse transcriptase (Promega). The mixture was incubated at 25 °C for 10 min, followed by 37 °C for 1 h. To synthesize second-strand cDNA/DNA, the reaction mixture was boiled for 2 min and cooled rapidly on ice, followed by incubation at 37 °C for 30 min in the presence of 5 U Klenow fragment (New England Biolabs, Ipswich, MA, USA) and 10 pmol of universal primer-dN6. Polymerase chain reaction (PCR) was conducted using a universal primer (5’-GCCGGAGCTCTGCAGAATTC-3’). PCR products larger than 500 bp in length were purified using an E.Z.N.A Gel Extraction Kit (Omega Bio-Tek, Norcross, GA, USA) and cloned into the pGEM-T Easy Vector (Promega). Recombinant plasmids were sequenced on an ABI Prism 3730 DNA Analyzer (Applied Biosystems, Foster, CA, USA) using the primer pair of M13 forward and reverse primer.

### 4.5. Full Genome Sequencing

Based on the sequences obtained by random PCR, the full-length sequence of the viral genome was acquired by PCR using specific primers (Table S1 in supplementary files). The 5’ and 3’ end sequences of the genome were obtained using a RACE kit (Takara, Dalian, China).

### 4.6. Phylogenetic Analysis

Routine sequence management and analyses were performed using DNAStar (DNAStar Inc., Madison, WI, USA). Using the Open Reading Frame Finder and GeneMark (version 2.8a), ORFs were predicted and identified by a translated BLAST search (BLASTx at http://www.ncbi.nlm.nih.gov/blast/Blast.cgi). Sequence alignment was performed using ClustalW and corrected manually [20]. The phylogenetic trees based on the full-length genomic sequences or deduced amino acid sequences of capsid proteins of all available dicistroviruses were constructed via the neighbor-joining (NJ) method with the MEGA program (version 6) with a bootstrap of 1000 replicates [21]. Gaps were regarded as a complete deletion.

### 4.7. Experimental Infection

One gram of moribund larvae was homogenized in 10 mL of TN buffer. After centrifugation at 3000× *g* for 5 min, the supernatant was filtered (0.45 μm) and diluted serially with TN buffer (1:100, 1:200, and 1:400) for infection. Three groups of healthy larvae (200 for each group) were immersed in the corresponding viral suspensions for 10 min in a volume of 500 mL and subsequently transported to 50 × 38 × 23 cm^3^ disinfected tanks. Control groups were treated with PBS. All of the tested or control larvae were fed *chirocephalus* three times per day. All the tanks were aerated gently and its water temperature was maintained at 28 to 30 °C. Excreta and food remains were removed and one-third of the freshwater was exchanged each day. Clinical signs and mortality were monitored daily. The dead larvae were collected and stored at −70 °C for detection.

### 4.8. RT-PCR Assay

RT-PCR with a specific primer pair was performed to detect the novel virus. Reverse transcription was performed in a 20 μL volume consisting of 6 μL of RNA and 10 pmol of random primer (dN_6_). The mixture was firstly denatured at 70 °C for 10 min, immediately quenched on ice, and subsequently added to the RT mixture containing 0.6 mM aliquots of each dNTP, 16 U RNasin (BioStar) and 200 U M-MLV reverse transcriptase (Promega). The reverse transcription reaction was conducted at 37 °C for 60 min, followed by heating to 70 °C for 5 min and holding at 4 °C.

For the amplification, the 25-μL reaction mixture contained 2 μL of cDNA, 2.5 μL of PCR buffer, 20 pmol of primer pair (MrTV472F: 5′-TGCTTCTATTTCGGCTCG-3′ and MrTV472R 5′-CAACGAATTAGGGAGAGG-3′), 0.2 mM dNTP, and 0.5 U Taq DNA polymerase (Promega). After an initial incubation step at 95 °C for 2 min, 35 cycles of amplification were carried out, consisting of denaturation at 94 °C for 30 s, annealing at 58 °C for 30 s, and extension at 72 °C for 40 s and a final extension step at 72 °C for 10 min. The expected products were gel purified by an E.Z.N.A. gel extraction kit (Omega Bio-Tek, Norcross, GA, USA) and sequenced for confirmation.

### 4.9. Nucleotide Sequence Accession Numbers

The genome sequence for MrTV was submitted to GenBank under accession No. HQ113110 or NC 018570.

## Supplementary Materials

Supplementary materials can be found at http://www.mdpi.com/1422-0067/17/2/204/s1.
