# Supplementary material for: Isolation and Characterization of a Novel Dicistrovirus Associated with Moralities of the Great Freshwater Prawn, Macrobrachium rosenbergii
Source: Int J Mol Sci. 2016 Feb 2;17(2):204. doi: 10.3390/ijms17020204 (PMC4783938; doi:10.3390/ijms17020204)
Supplement: Supplementary file 1 [file ijms-17-00204-s001.pdf]

Supplementary Materials: Isolation and Characterization of a Novel Dicistrovirus Associated with Moralities of the Great Freshwater Prawn, *Macrobrachium rosenbergii*

Xiaoyi Pan, Zheng Cao, Junfa Yuan, Zhengli Shi, Xuemei Yuan, Lingyun Lin, Yang Xu, Jiayun Yao, Guijie Hao and Jinyu Shen

Table S1. Oligonucleotide primers used for full genome sequencing.

Oligonucleotide Designation	Reaction	Oligonucleotide (5′→3′)	Genome Position	Orientation
R517	5′ RACE	GTCGGAATCTTGTTCGGTAATC	517–496	←
R1021	5′ RACE	TTCAAGCCAGGCACCAATAA	1021–1002	←
F1044	Gap-filling	ACAGTGCGGCTTCACCTAAA	1044–1063	→
R2061	Gap-filling	CTACGCATCCCAACCAAAGC	2061–2042	←
F2441	Gap-filling	TATGGAGATGCTGGATGTGG	2441–2460	→
R3273	Gap-filling	TTCAGGGACTCGGTCAGAT	3273–3255	←
F3222	Gap-filling	GCTTTGCTACTGACTGGTTG	3222–3241	→
R4813	Gap-filling	CTTTGCTTCAAAACCTTGCC	4813–4794	←
F5321	Gap-filling	GGATTAGAAGGGCACCAGTAT	5321–5341	→
R5970	Gap-filling	GGATTACCTGATGGTTGAGAG	5970–5950	←
F5686	Gap-filling	TGAAATTGGTTTGGGAGT	5686–5703	→
R6238	Gap-filling	AGAGCGATAAGGAGGTGG	6238–6221	←
F6382	Gap-filling	ACGGATTGCTTCGTTTGA	6382–6399	→
R7749	Gap-filling	TTGGAGCTTGCTAAGGACTG	7749–7730	←
F8549	3′ RACE	TTCTGCTGTTGATGATAAGGG	8549–8569	→
RACEdT	3′ RACE	GCCGGAGCTCTGCAGAATTCT$_{17}$VN	/	/
BRS	3′ RACE	GCCGGAGCTCTGCAGAATTC	/	/

→: forward; ←: reverse.

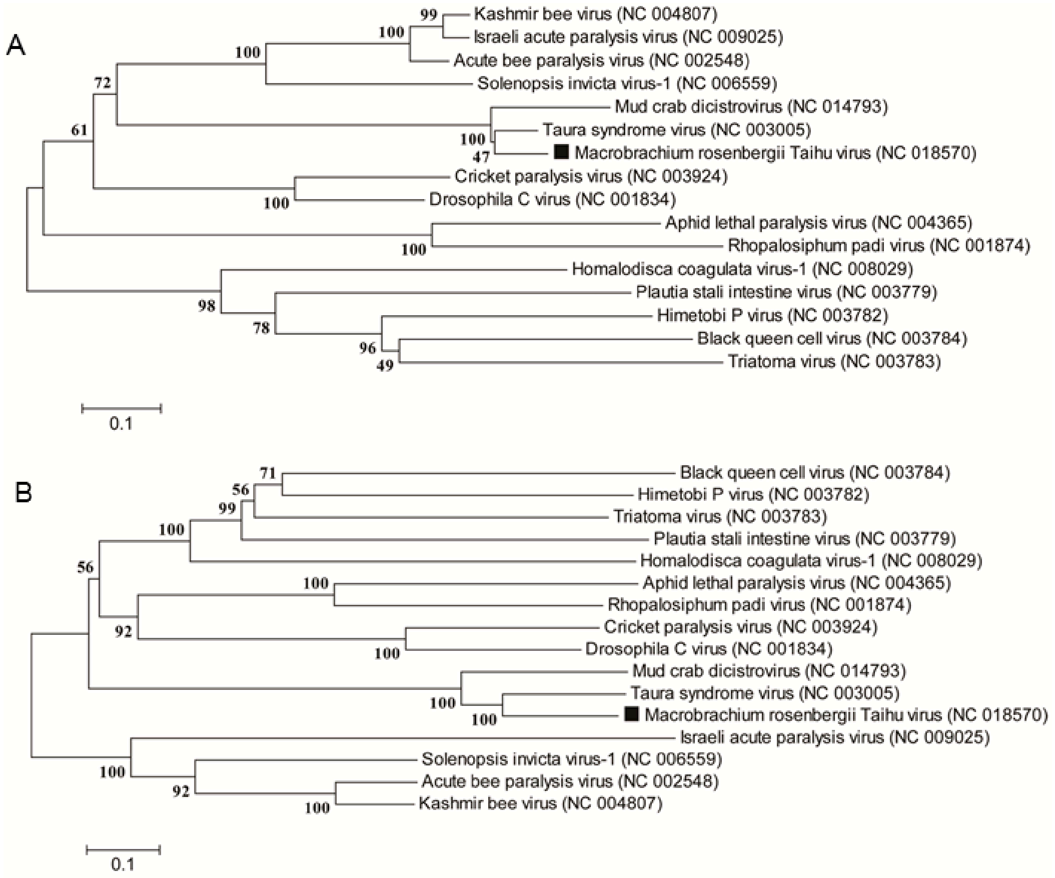

Figure S1. Phylogeny of the MrTV and other dicistroviruses. Phylogenetic trees based on the other the deduced amino acid sequence of RNA-dependent RNA polymerase (RdRp) (**A**) and replicase polyprotein (**B**) were constructed using the neighbor-joining method with 1000 bootstrap replicates under the parameter of complete deletion and Jones-Taylor-Thornton (JTT) model using MEGA 6.0. The numbers at the branch nodes represent the bootstrap confidence levels of the 1000 bootstrap replications obtained. Bar, 0.1 amino acid substitutions per site. The black aquares indicate MrTV.
